# Comparative iTRAQ proteomics revealed proteins associated with lobed fin regeneration in Bichirs

**DOI:** 10.1186/s12953-019-0153-0

**Published:** 2019-11-20

**Authors:** Suxiang Lu, Qian Xiong, Kang Du, Xiaoni Gan, Xuzhen Wang, Liandong Yang, Ying Wang, Feng Ge, Shunping He

**Affiliations:** 1 Key Laboratory of Aquatic Biodiversity and Conservation of the Chinese Academy of Sciences, Institute of Hydrobiology, Chinese Academy of Sciences, Wuhan, 430072 Hubei China; 2 Present address: Medical College of Pingdingshan University, Pingdingshan, 467000 Henan Province China; 3 Key Laboratory of Algal Biology, Institute of Hydrobiology, Chinese Academy of Sciences, Wuhan, 430072 Hubei China

**Keywords:** Quantitative proteome, *Polypterus senegalus*, Limb, Regrowth, Blastema

## Abstract

**Background:**

*Polypterus senegalus* can fully regenerate its pectoral lobed fins, including a complex endoskeleton, with remarkable precision. However, despite the enormous potential of this species for use in medical research, its regeneration mechanisms remain largely unknown.

**Methods:**

To identify the differentially expressed proteins (DEPs) during the early stages of lobed fin regeneration in *P. senegalus*, we performed a differential proteomic analysis using isobaric tag for relative and absolute quantitation (iTRAQ) approach based quantitative proteome from the pectoral lobed fins at 3 time points. Furthermore, we validated the changes in protein expression with multiple-reaction monitoring (MRM) analysis.

**Results:**

The experiment yielded a total of 3177 proteins and 15,091 unique peptides including 1006 non-redundant (nr) DEPs. Of these, 592 were upregulated while 349 were downregulated after lobed fin amputation when compared to the original tissue. Bioinformatics analyses showed that the DEPs were mainly associated with Ribosome and RNA transport, metabolic, ECM-receptor interaction, Golgi and endoplasmic reticulum, DNA replication, and Regulation of actin cytoskeleton.

**Conclusions:**

To our knowledge, this is the first proteomic research to investigate alterations in protein levels and affected pathways in bichirs’ lobe-fin/limb regeneration. In addition, our study demonstrated a highly dynamic regulation during lobed fin regeneration in *P. senegalus*. These results not only provide a comprehensive dataset on differentially expressed proteins during the early stages of lobe-fin/limb regeneration but also advance our understanding of the molecular mechanisms underlying lobe-fin/limb regeneration.

## Background

The mystery of limb regeneration in vertebrates has attracted the attention of scientists for many years. The newts and salamanders have one iconic capacity of complete regeneration of all appendages [1]. Anurans have only restricted regeneration capacity, and usually lose it after metamorphosis [2]. Mammals have the capacity of regeneration of the digit tip, but not limb [3]. *Polypterus* regenerates its lobed pectoral fins with remarkable accuracy [4]. Advances in the study of the development of regenerated limbs have been reported, but why certain species are able to regenerate their limbs is still unknown. In addition, the molecular mechanisms in bichirs lobed fins (endoskeleton) regeneration process remain unknown. It is possible that if we comprehensively analyze the molecular mechanisms involved in the regeneration of lobed pectoral fins in bichirs, we can more easily understand the process of complete lobe-fin/limb regeneration ability.

Salamanders seem to be the only living tetrapods that can regenerate full limbs [5]. However, among vertebrates, the paired lobe-fins/limbs of adult lungfishes and bichirs are also able to fully regenerate [4, 6, 7]. It is notable that the lobe-fins/limbs of bichirs include a bony endoskeleton that differs from the dermal exoskeleton of the fins of teleost fishes, although bichirs were always classified as Actinopterygii [8]. In addition, all three of these species live in water-land transitional environments.

*Polypterus senegalus,* also known as the Senegal bichir or the gray bichir, is a prototypical fish species in the *Polypterus* genus. Although the oldest fossil polypteriformes records are only known from 112 to 99 Mya [9], some phylogenetic hypotheses predict a divergence age of 385 to 426.8 Mya [8, 10]. *Polypterus* shares many characteristics with amphibians in body structure, such as paired lungs [11–14]. In addition, it has been observed crawling on land both in the lab [15] and in the open [16] by its complex lobed fins, which have equivalent radius, ulna, radiale and ulnare [4, 14, 17].

Newt is remarkable in terms of its regenerative ability. However, it is not as convenient as other model organisms. To some degree, such a phenomenon is caused by their enormous genome size, about 25–50 pg (C value) that is approximately 10 times of that of humans (C value 3.5 pg). In addition, the large introns in newt genes may also reduce the value of this group for research compared to other tetrapods [18]. Previous studies have found that *P. senegalus* is able to initiate lobe-fin/limb regeneration similar to amphibian, with the formation of a blastema [4, 19]. *P. senegalus* has a relative small genome size (~ 3.4 Gbp), and short time of reproductive cycle (could be as short as 6 months) [11], making it a better candidate as a model organism for limb/lobe-fin regeneration in vertebrates. Polypteriformes are characterized with derived and plesiomorphic features, which make these fishes attractive subjects for evolutionary and developmental comparisons of lobe-fin/limb regeneration. The research herein was targeted at identifying candidate genes and molecular mechanisms that might have played a vital role in lobe-fin/limb regeneration.

## Results

### Growth curve

**Fig. 1:**
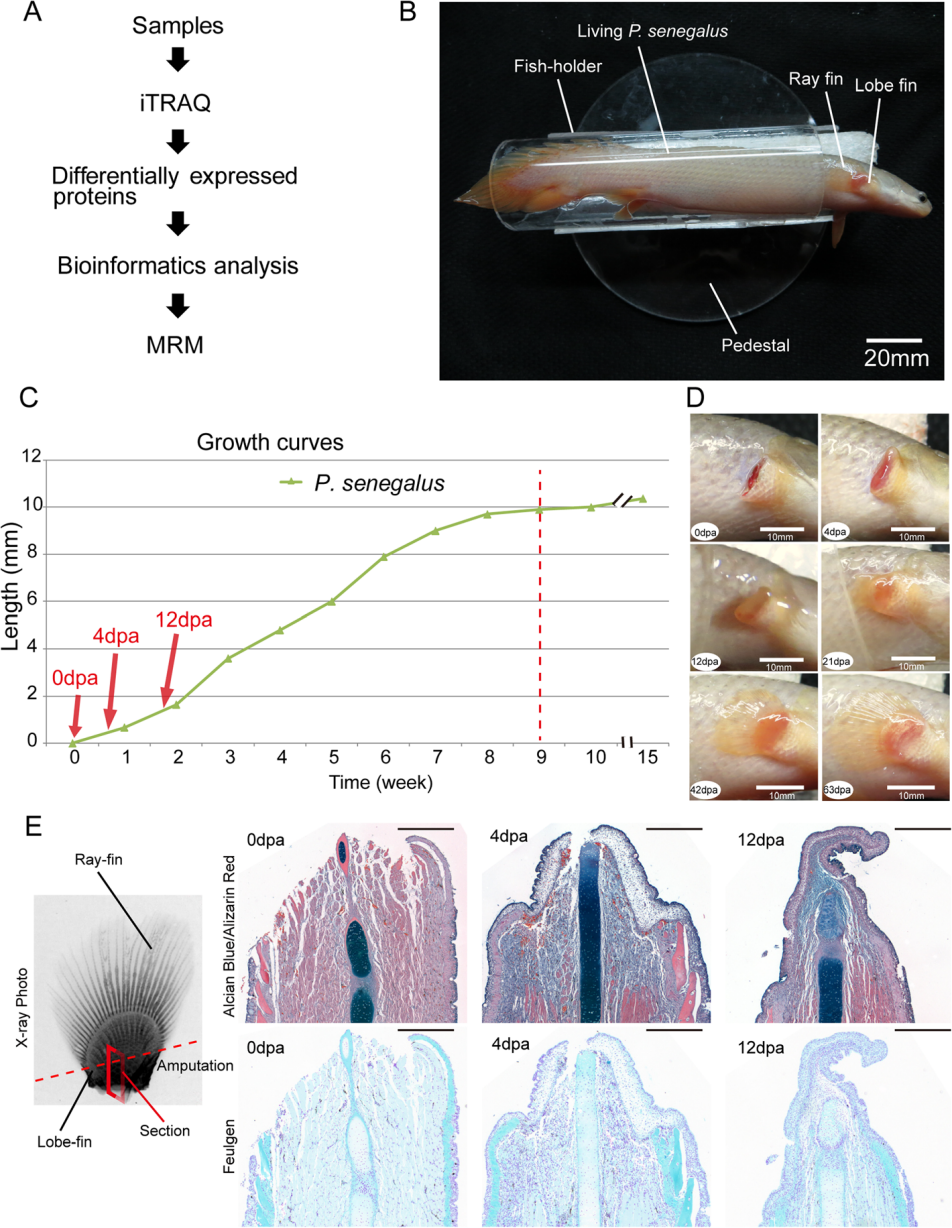
Study sampling and experimental scheme. **a** The overall workflow of this study. **b** A photo of a live specimen of *P. senegalus* with a homemade fish-holder. **c** Growth curve of *P. senegalus* pectoral lobed fin after amputation. The sampling time points of 0 dpa (0D_L and 0D_R), 4 dpa (4D_B) and 12 dpa (12D_D) are labeled. **d** *P. senegalus* lobed fins at different regeneration stages. **e** Tissues of three time points were shown by Alcian blue- and alizarin red- staining, and feulgen staining on paraffin tissue sections. The position of the section was shown on the X-ray photo. Scale bars: 0.5 mm. dpa: days post amputation

The overall workflow and animal sample for the present study were displayed in Fig. 1a and b, respectively. The green curve in Fig. 1c represents the growth of the regenerating forelimbs of *P. senegalus* over 15 weeks, and the sampling time points of 0 dpa (0D_L and 0D_R), 4 dpa (4D_B) and 12 dpa (12D_D) are marked. The regenerating forelimbs of *P. senegalus* exhibited a rapid growth rate in the early stage, and after 9 weeks, it plateaued and persisted for a long time. During the plateaued stage, the growth rate was low (only 0.35 mm over 5 weeks). Photos of regenerating lobed fins at key time points were obtained (Fig. 1d). In order to provide an intuitive understanding of the lobed fin regeneration process, the histologic features of stump tissue in 0 dpa, 4 dpa, and 12 dpa were shown in Fig. 1e.

### Overview of the library and quantitative proteomics analysis

An 8-label isobaric tags for relative and absolute quantification (iTRAQ) experiment was performed for eight samples of the pectoral fins of bichirs at three time points, using *P. senegalus* genome data as background library. The overall proteomic results, such as basic descriptive statistics, peptide length distribution, protein coverage distribution, and mass errors of all of the identified peptides, are presented in Additional file 1. A total of 3177 proteins and 15,091 unique peptides were quantified in our experiment (Additional file 2), representing 15.4% of the 20,582 predicted proteins in the *P. senegalus* genome. We determined that 1006 non-redundant (nr) proteins were differentially regulated during the regeneration progress. Among these proteins, 592 (4D_B/0D_L) and 87 (12D_D/4D_B) were upregulated while 349 (4D_B/0D_L) and 55 (12D_D/4D_B) were downregulated after lobed fin amputation (Additional file 3).

Comparison of replicate datasets—Using the quantitative value of proteins in the duplicate data, the degree of variation and the distribution of statistical variation were calculated. All of the mean CV values were less than 0.15, revealing a relatively high repeatability among multiple samples (Additional file 4: A), and the protein ratio distribution is shown in Additional file 4: B. On the basis of protein ratios transformed by log2, we conducted a linear regression analysis to compare the two experimental replicates, so as to evaluate the reproducibility of the iTRAQ proteomic results. R^2^ values showed the strong linear correlation of the two experimental replicates for both 4D_B and 12D_D (Additional file 4: C). As indicated by these results, the level of reproducibility between replicate datasets was high.

### Functional classification of differentially expressed proteins

**Fig. 2:**
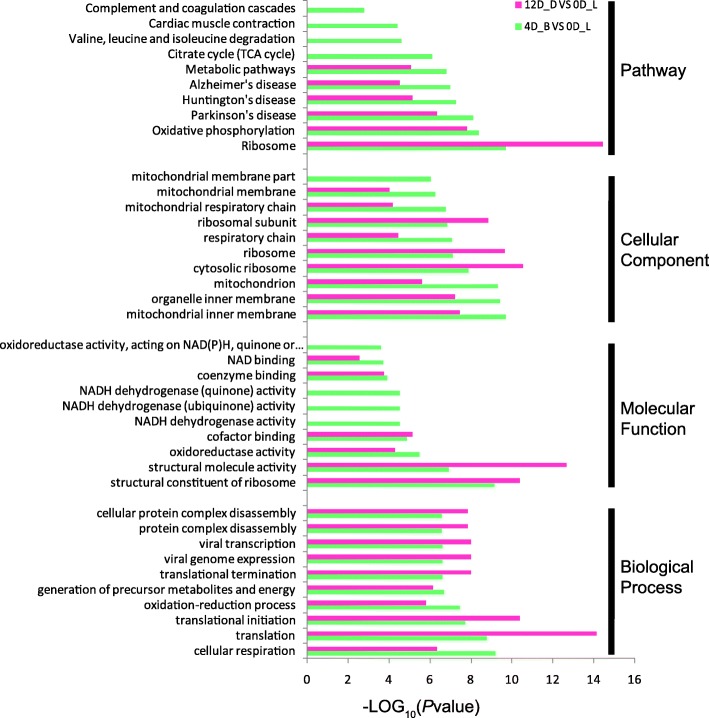
Enrichment analysis of the differentially expressed proteins in 4D_B and 12D_D. GO terms and KEGG pathways enriched in the differentially expressed proteins at 4 and 12 days post amputation. 12D_D VS 0D_L was shown in red. 4D_B VS 0D_L was shown in green

It was shown from Fig. 2 (only *p* < 0.001 and top 10 terms were showed) that at both 4D_B and 12D_D, five KEGG pathways were regulated differently, which was displayed in the analysis result of KEGG pathway enrichment: “Ribosome” (ko03010), “Oxidative phosphorylation” (ko00190), “Parkinson’s disease” (ko05012), “Huntington’s disease” (ko05016), “Alzheimer’s disease” (ko05010) and “Metabolic pathways” (ko01100) (Additional file 5: A).

After GO term enrichment analysis, we got several biological processes, including “cellular respiration translational termination”, “translation”, “translational initiation”, “oxidation-reduction process” and “generation of precursor metabolites and energy” were significantly enriched in both 4D_B and 12D_D, suggesting that the processes above are very active during the regeneration process (Additional file 5: B).

The GO molecular function terms, including “ribosome structural constituent,” “structural molecule activity,” “oxidoreductase activity” and “cofactor binding,” and GO cellular component terms, including “mitochondrial inner membrane,” “organelle inner membrane,” “mitochondrion,” “cytosolic ribosome,” “ribosome” and “respiratory chain,” were significantly enriched in both 4D_B and 12D_D (Additional file 5: C and D).

### Protein−protein interaction network analysis

**Fig. 3:**
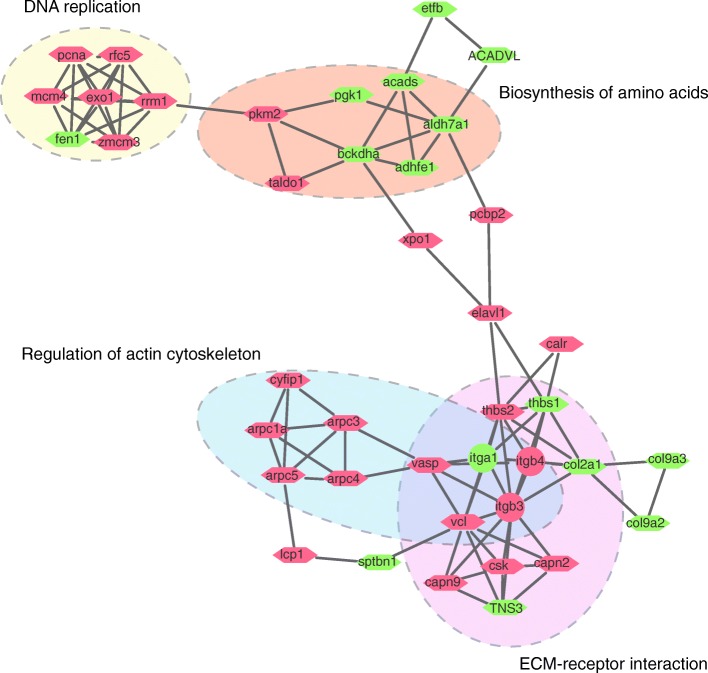
Protein-protein interaction network of DEPs in cluster 7 by STRING and MCODE. Proteins that are associated with each other are linked by an edge. The up-regulated DEPs (4D_B/0D_L) in 4D_B sample were in red, and the down-regulated DEPs were in green. Integrins were displayed in circles. The main pathway descriptions of these DEPs were from STRING

To further understand the biological process or pathway regulated in the early stage of lobe fin regeneration, a protein interaction network of DEPs in 4D_B/0D_L was performed by STRING with *Xenopus Silurana* as background organism. As a result, 697 nodes and 8263 edges were retrieved. After the computing by MCODE in Cytoscape, 16 clusters were obtained (Additional files 6 and 7). And the top 10 clusters were displayed here. Cluster 1 and 9 consists of proteins involved in ribosome and RNA transport. Cluster 2–6 consists of proteins involved in oxidative phosphorylation and metabolic pathways. Cluster 7–8 consists of proteins involved in ECM-receptor interaction. Cluster 10 consists of proteins involved in Golgi and endoplasmic reticulum. It is worth noting that cluster 7 contains three integrins, and the main signaling pathways involved are DNA replication, Biosynthesis of amino acids, Regulation of actin cytoskeleton, and ECM-receptor interaction (Fig. 3).

### Validation of changes in protein expression using multiple-reaction monitoring analysis

**Fig. 4:**
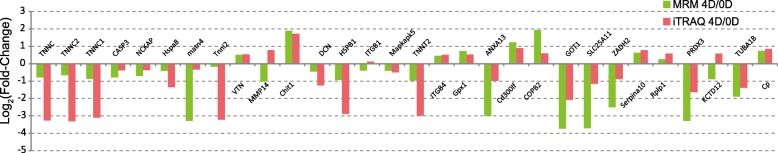
Validation of some proteins. Correlation between MRM and iTRAQ data of 30 proteins. 4D/0D, 4 days post amputation / 0 day post amputation

Multiple-reaction monitoring (MRM) was used to provide further independent validation of the expression status of 30 of the quantified proteins. Finally, 27 results from MRM were in line with the quantitative proteomic results (Fig. 4). Details of the dynamic MRM results, including the fold changes, normalized intensities and *p*-values of 30 proteins are given in Additional file 8.

## Discussion

### The lobe fin regeneration proteome

This study, for the first time, obtained a quantitative proteome profile of lobed fin (endoskeleton) regeneration in bichirs. The growth curve in *P. senegalus* (Fig. 1c) shows a difference with the growth curve in *N. viridescens* [20]. Iten and Bryant’s research on newt shows that amputation location information does not affect the developmental sequence of limb regeneration events, but it does affect the rate of growth of regenerates [20]. Although the lengths of the resections were different, their amputation positions were located at the proximal end of the ulna along the longitudinal axis. Therefore, it is still speculated that the growth rate of bichir appendage is higher in the early stage than the newt limb.

The *P. senegalus* limb/lobe-fin regeneration proteome contains proteins that are expressed in the cancer stem cells, nerve cells, muscle cells, and cartilage cells. As a consequence, we found cancer stem cell-associated proteins, such as mmp2, mmp9, mmp13, mmp14, paxillin, Ras, src and c-myc [21, 22], as well as genes expressed in the immune system, such as C5 [23], C9 [24], and HMGB1 [25]. Besides, numerous renowned and supposed growth regulation proteins were detected. Among those were c-met [26], FGF2 [27] and TGFBI [28]. We also identified a MARCKS-like protein (MLP) that was previously reported to induce the initial cell cycle response during limb regeneration in axolotls [29]. Furthermore, considerable proteins that were reported to be engaged in neurodegeneration mechanisms during limb regeneration were found, such as CASP3 [30], CASP7 [31], and its related proteins ERK1/2 [32], Clathrin [33], Cdk5 [34], SOD2 [35], and ApoE [36, 37]. In addition to stem cell pathways, we also identified wnt/β-Catenin signaling proteins which are reported to play an important role in regulating vertebrate limb regeneration, such as GSK-3β, mTOR and the β-Catenin [38, 39]. As demonstrated from these results, within the first 12 days, a wide variety of protein families that have different functions expressed in the epimorphosis process of the lobed fin are included in this proteome.

### Blastema formation

In a recent study, through the iTRAQ method, Tang, J., et al. found that differentially expressed proteins were associated with wound healing, immune response, cellular process, metabolism and binding in the salamanders’ blastema formation [40]. By similar iTRAQ method, Geng et al. found that in the early stages of newt’s blastema formation, differentially expressed proteins were concentrated in the categories such as signaling, Ca^2+^ binding and translocation, transcription and translation, immune response, cell death, cytoskeleton, metabolism [41]. Correspondingly, in the current study, DEPs are mainly concentrated in ribosome and RNA transport, metabolic, ECM-receptor interaction, Golgi and endoplasmic reticulum, DNA replication, and Regulation of actin cytoskeleton, suggesting that these biological processes are active during the appendage regeneration process.

Integrin signaling plays an indispensable role in the regeneration process of the spinal cord [42]. In the current study, we found that the integrins-related signaling pathway was significantly enriched in bichirs lobe-fin regeneration. It was demonstrated that αvβ5 integrin plays a key role in the dedifferentiation of chondrocytes by activating ERK signaling [43]. Previous studies have shown that integrin is involved in spontaneous axonal regeneration after peripheral nerve injury, and axonal regeneration would be inhibited if integrins were inactivated by axon-repulsive molecules [43–45]. It is concluded that the regeneration process in vivo is always associated with increased integrin [42]. However, our study found that during the blastema formation of bichirs, two integrins were up-regulated, but one integrin was down-regulated. This means that integrins may play a complex role in the process of appendage regeneration.

### Differentially regulated proteins during limb/lobe-fin regeneration

Using our iTRAQ method, hundreds of genes that were differentially regulated in various periods of limb/lobe-fin regeneration were identified. For instance, MMP13a and VTN were strongly upregulated during the first four days. MMP13 has been implicated as a contributor to skeletal muscle regeneration and is critical for myoblast migration [46], and VTN is a regulator of multimerization and collagen binding during liver regeneration [47], as well as a growth factor complex for wound repair and tissue regeneration [48]. It is possible that MMP13a and VTN also play an important role in the regulation of lobed fin regeneration in bichirs. We also found many other kinds of endopeptidases, showing that the remodeling of the extracellular matrix is essential for regeneration.

Another gene from the same cluster, Chit1, which is a biochemical marker of macrophage activation [49], was strongly upregulated during the first four days. Macrophages remove tissue debris and activate stem cell populations [50] and are required for adult salamander limb regeneration [51].

The decorin protein gene DCN was downregulated during the first four days but was later upregulated in the days that followed, and this expression pattern is consistent with this gene playing two different roles during regeneration. During the early stages, DCN may be involved in the response to dedifferentiation because a low level of decorin is conducive to the growth of various tumor cell lines and to an increased abundance of anti-inflammatory molecules [52]. During the later stages, DCN may be involved in the response to redifferentiation and growth because a high level of decorin promotes muscle cell differentiation and muscle regeneration [53], suppresses scar formation and promotes axon growth [54]. Finally, it is possible that the level of expression of decorin play a key role in lobe-fin/limb regeneration.

HSPB1 (HSP27) is a protein that is related to regeneration, which could speeds up axonal growth in vitro after peripheral nerve injury [55]. The HSPB1 protein was downregulated during the first four days but was later upregulated in the days that followed. During the early stages, the low level of HSPB1 may have occurred in response to apoptosis because HSPB1 is involved in protection against necrotic and apoptotic cell death. The high level of HSPB1 during the later stages was consistent with its late-expression property, which is different from most axonal injury-regulated and growth-associated genes [56].

Integrin beta 4 (ITGB4) is also called CD104. In the biology of invasive carcinoma, it is likely to play a crucial role. Integrins adjust cell-extracellular matrix (ECM) or cell-cell interactions, and transformed signals that manage the growth of cell and expression of gene. The forced activation of integrins can overcome inhibition and increase axon regeneration [57]. Integrin-beta 1 (ITGB1) regulates chondrocyte proliferation and apoptosis [58], and the knockdown and knockout of ITGB1 in hepatocytes impair liver regeneration [59]. The ITGB4 protein was upregulated during the first four days and later returned to normal levels in the days that followed. During the early stages, ITGB4 may be involved in the response to dedifferentiation and cell migration because the upregulation of ITGB4 can restore the regenerative performance of adult neurons [60], and promote epithelial-mesenchymal transition, cell scattering, cell motility, and vimentin expression [61].

Membrane transporters, synaptic transmission, and regulators of nerve development were found in the considerable enrichment, supporting a model in which nerve signaling might be crucial for early stages of limb regeneration. As shown from recent researches, the regeneration polarity in planarians can be regulated by the signaling of ventral nerve cords [62]. And limb regeneration in axolotl can be enhanced by nerve signaling [63, 64].

**Fig. 5:**
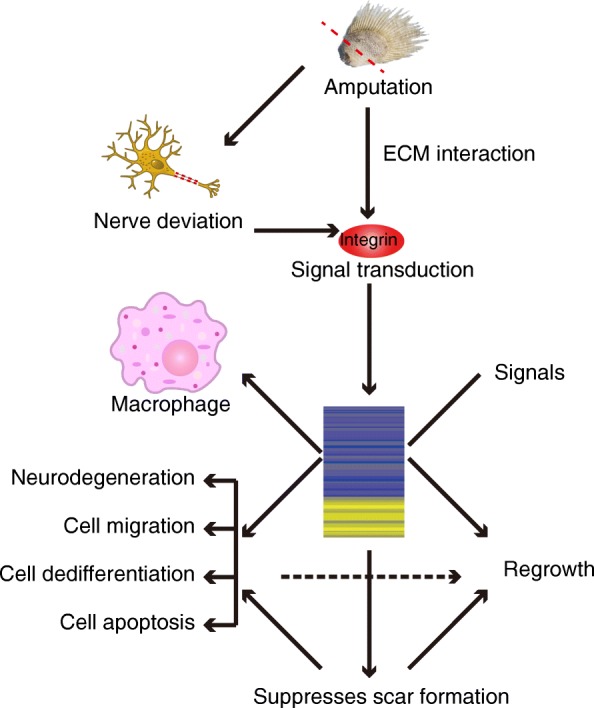
Proposed model depicting the molecular mechanism based on the *P. senegalus* proteome. The combination of all of these mechanisms regulates the expression of hundreds of proteins and promotes cell migration, dedifferentiation and regrowth, and scar suppression

In summary, as revealed from our time-course analysis, the limb/lobe-fin regeneration is regulated by a dynamic, complicated proteomic network that is rapidly caused after amputation (Fig. 5), which might be triggered by nerve deviation and ECM interaction and transducted by integrins that launch a cascade of regenerative processes, such as cell migration, dedifferentiation and regrowth, as well as scar suppression.

### Validation of the proteome result

To validate the results of the proteome obtained from the current study, we used the Multiple reaction monitoring (MRM) approach. Multiple reaction monitoring (MRM) using mass spectrometry is a highly sensitive and selective method for the targeted quantitation of protein/peptide abundances in complex biological samples. Previous studies have shown that targeted MRM proteomics can be used as a verification tool for candidates in the context of a comprehensive proteome [65, 66]. Among the 30 selected target proteins, the expression trend of 27 proteins was verified, and the difference in numerical values may be attributed to differences in statistical models or biological variation between the samples. Three candidate proteins, such as MMP14, ITGB1, and KCTD12, show opposite expression trends in iTRAQ and MRM results, which may be due to biological variation between the samples.

## Conclusions

In this study, we constructed a lobed fin regeneration proteome for *P. senegalus* and generated time-resolved profiles of the expressed genes based on the growth curve. Functional analysis indicated that the differentially expressed proteins were associated with Ribosome and RNA transport, metabolic, ECM-receptor interaction, Golgi and endoplasmic reticulum, DNA replication, and Regulation of actin cytoskeleton. These results not only provide a comprehensive dataset on differentially expressed proteins during the early stages of lobe-fin/limb regeneration but also advance our understanding of the molecular mechanisms underlying lobe-fin/limb regeneration.

## Methods

### Tissue collection and image acquisition

The animal experiment for this study was approved by the Institutional Animal Care and Use Committee of Institute of Hydrobiology (Approval ID: Y21304501). Adult specimens of *P. senegalus* were commercially obtained and maintained in glass tanks at 26 °C. The bichirs were anesthetized in 0.1% Tricaine mesylate (MS-222) before image collection and sampling. The bichirs were then washed with pure water and dried with filter paper to clean the pectoral fins, and amputations were performed with small surgical scissors and forceps along the cross section of the middle of the base of the pectoral lobe fin. For iTRAQ, tissues (or regenerates) were collected at 0, 4 and 12 days post-amputation (dpa), and these experiments were conducted in two biological replicates (distinguished by _C and _E), with each replicate consists of 10-pooled biological tissues for each stage. The 0 dpa sample was from the amputated fin itself, and divided into the 0 dpa lobe-fin (0D_L) sample and the 0 dpa ray-fin (0D_R) sample. The 4 dpa sample which consists of blastema was named 4D_B. The 12 dpa sample which consists of re-differentiation tissues was named 12D_D. The samples were flash frozen by liquid nitrogen for protein preparation. For MRM, the same method was used to collect tissues (or regenerates), with three replicates.

The regeneration progress was observed to develop the lobed fins regeneration growth curve. A vernier caliper and a camera were used to measure the length of the regenerates and to take photos, respectively, over 15 weeks. For histological analysis, stump tissues of 0 dpa, 4 dpa, and 12 dpa were collected and immersed in the 4% paraformaldehyde fixative.

### iTRAQ process and MRM process

iTRAQ process—The tissues were treated using the same method as previously described [67]. Briefly, the tissues were split and then the proteins were reduced. Then, Bradford method was used to determine the protein concentration. Total protein was digested and processed with 8-plex iTRAQ reagent (Applied Biosystems) as previously described with minor modifications [67]. Briefly, the samples were labeled with the iTRAQ tags as follows: Sample 0D_R_C (117 tag), Sample 0D_R_E (113 tag), Sample 0D_L_E (114 tag), Sample 0D_L_C (118 tag), Sample 4D_B_E (115 tag), Sample 4D_B_C (119 tag), Sample 12D_D_E (116 tag), and Sample 12D_D_C (121 tag).

To separate the iTRAQ-labeled peptide mixtures, load sample and acquire data, the same method was used as previously described [67]. Briefly, peptide supernatant was loaded into an LC-20 AD nano HPLC and then eluted onto a 10 cm analytical C18 column. Data acquisition was performed with a Triple TOF 5600 System fitted with a Nano spray III source and a pulled quartz tip as the emitter. The raw data files acquired from 5600 were converted into MGF files using 5600-ms converter, and the MGF files were searched. Protein identification was performed using the Mascot search engine (Matrix Science, London, UK; version 2.3.02) against the *P. senegalus* genome database containing 20,582 sequences.

Proteins were identified and quantified as previously described with minor modifications [68]. Briefly, peptides with significance scores (≥20) at the 99% confidence interval by a Mascot probability analysis greater than “identity” were counted as identified. Only *p* values < 0.05, fold changes larger than 1.4 were considered significant differential expressions of proteins.

MRM process—For protein digestion, the same method was used as in iTRAQ process. Then, the samples were spiked with 50 fmol of β-galactosidase for data normalization, and MRM analyses were performed on a QTRAP 5500 mass spectrometer (AB SCIEX, Foster City, CA) equipped with a Waters Nano-Acquity Ultra Performance LC system. The method was as previously described with minor modifications [69]. Briefly, the peptides were separated on a BEH130 C18 column (0.075 × 200-mm column, 1.7 μm; Waters) at 300 nL/min and eluted with a gradient of 2–40% solvent B for 30 min, 40–60% solvent B for 3 min, a 2-min linear gradient to 80% solvent B and maintenance at 80% for 5 min. For the QTRAP 5500 MS, a spray voltage of 2100 V, nebulizer gas at 20 psi, and dwell time of 10 ms were used. We use MSstats with the linear mixed-effects model the *P* values were adjusted to control the FDR at a cutoff of 0.05. After data analysis with Skyline software, all of the proteins with a *p* value < 0.05 and a fold change > 1.5 were considered significant.

### Bioinformatics analysis

Functional annotations of the proteins were conducted using Blast2GO [70] program against the non-redundant protein database (NR; NCBI; v2.5; animal; 8,428,593 sequences). Orthologous proteins were clustered based on the Cluster of Orthologous Groups (COGs) of proteins database [71]. KEGG (animal) [72] was used to identify the molecular interaction and reaction networks of these proteins. Hypergeometric tests were employed to perform GO enrichment and KEGG pathway enrichment, with reference dataset of all identified proteins in the whole proteome. The protein interaction network analysis was conducted using STRING (http://string-db.org/). Then the network was visualized by Cytoscape v3.2.1 and further analyzed for densely connected regions by Molecular Complex Detection (MCODE) v1.4.1 [73].

## Supplementary information

**Additional file 1.** All of the proteins identified in the present study with annotations.

**Additional file 2.** Overview of the proteomic results. (A) Basic information statistics. (B) Identified peptide distribution. (C) Protein coverage. (D) Mass errors were determined for all identified peptides.

**Additional file 3.** Differentially expressed proteins at different time points.

**Additional file 4.** Congruence of evaluation among different data set. (A) CV distribution. a, 0D_L-VS-4D_B. b, 0D_L-VS-12D_D. (B) Protein ratio distribution. c, 0D_L-VS-4D_B. d, 0D_L-VS-12D_D. (C) Linear regression analysis based on the log2-transformed protein ratios. e, linear correlation between the two experiment replicates for 4D_B. f, linear correlation between the two experiment replicates for 12D_D.

**Additional file 5.** GO and pathway enrichment analysis of differentially expressed proteins.

**Additional file 6.** Protein-protein interaction networks of DEPs in 4D/0D.

**Additional file 7.** Some protein-protein interaction clusters that calculated by the MCODE software.

**Additional file 8.** Details of the dynamic MRM results.

## Data Availability

All raw data of proteome has been deposited in the Peptide Atlas database (http://www.PeptideAtlas.org) with the accession PASS00842.

## Abbreviations

0D_L: 0 dpa lobe-fin sample
0D_R: 0 dpa ray-fin sample
12D_D: 12 dpa sample which consist of re-differentiation tissues
4D_B: 4 dpa sample which consist of blastema
ApoE: Apolipoprotein E
C5: Complement component 5
C9: Complement component 9
CASP: Caspase
Cdk5: Cyclin dependent kinase 5
Chit1: Chitotriosidase-1
COGs: Cluster of Orthologous Groups
CV: Coefficient of variation
DCN: Decorin
DEPs: Differentially expressed proteins
dpa: days post amputation
ECM: Extracellular matrix
ERK: Extracellular signal–regulated kinases
FGF: Fibroblast growth factor
GO: Gene ontology
GSK-3β: Glycogen synthase kinase 3 beta
HMGB: High mobility group box
HSPB1: Heat shock protein beta-1
ITGB4: Integrin Subunit Beta 4
iTRAQ: isobaric tag for relative and absolute quantitation
KEGG: Kyoto Encyclopedia of Genes and Genomes
MLP: MARCKS-like protein
MMP13a: matrix metallopeptidase 13a
mmp14: matrix metallopeptidase 14
mmp2: matrix metallopeptidase 2
mmp9: matrix metallopeptidase 9
MRM: Multiple-reaction monitoring
MS: Mass spectrometry
mTOR: mammalian target of rapamycin
Mya: Million years ago
Nr: Non-redundant
SOD2: Superoxide dismutase 2
TGFBI: Transforming Growth Factor Beta Induced
VTN: Vitronectin
