# Supplementary figures and images for: Comparative iTRAQ proteomics revealed proteins associated with lobed fin regeneration in Bichirs
Source: Proteome Sci. 2019 Nov 20;17:6. doi: 10.1186/s12953-019-0153-0 (PMC6869209; doi:10.1186/s12953-019-0153-0)

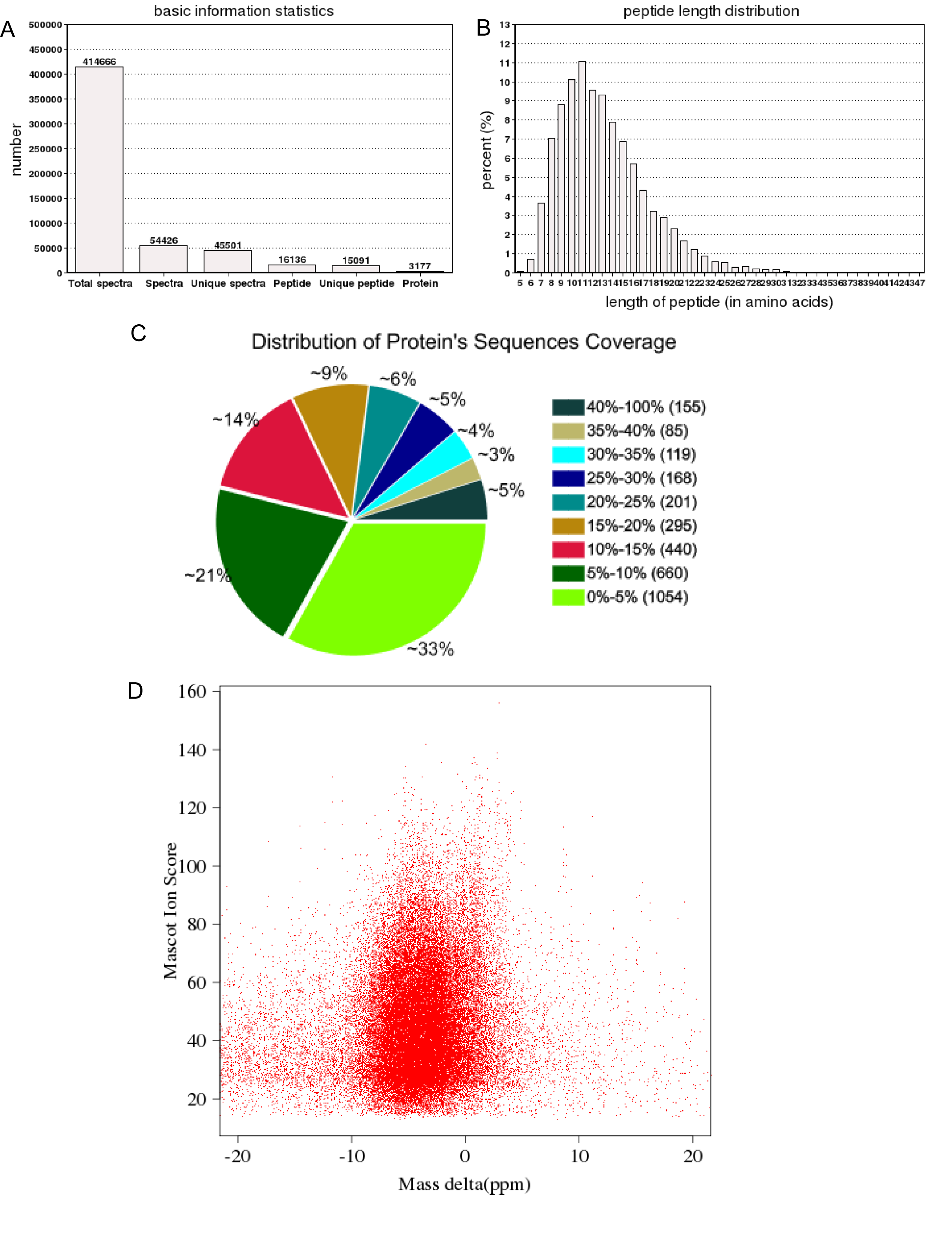

Supplement: Supplementary file 2 — Additional file 2. Overview of the proteomic results. (A) Basic information statistics. (B) Identified peptide distribution. (C) Protein coverage. (D) Mass errors were determined for all identified peptides. [file 12953_2019_153_MOESM2_ESM.tif]

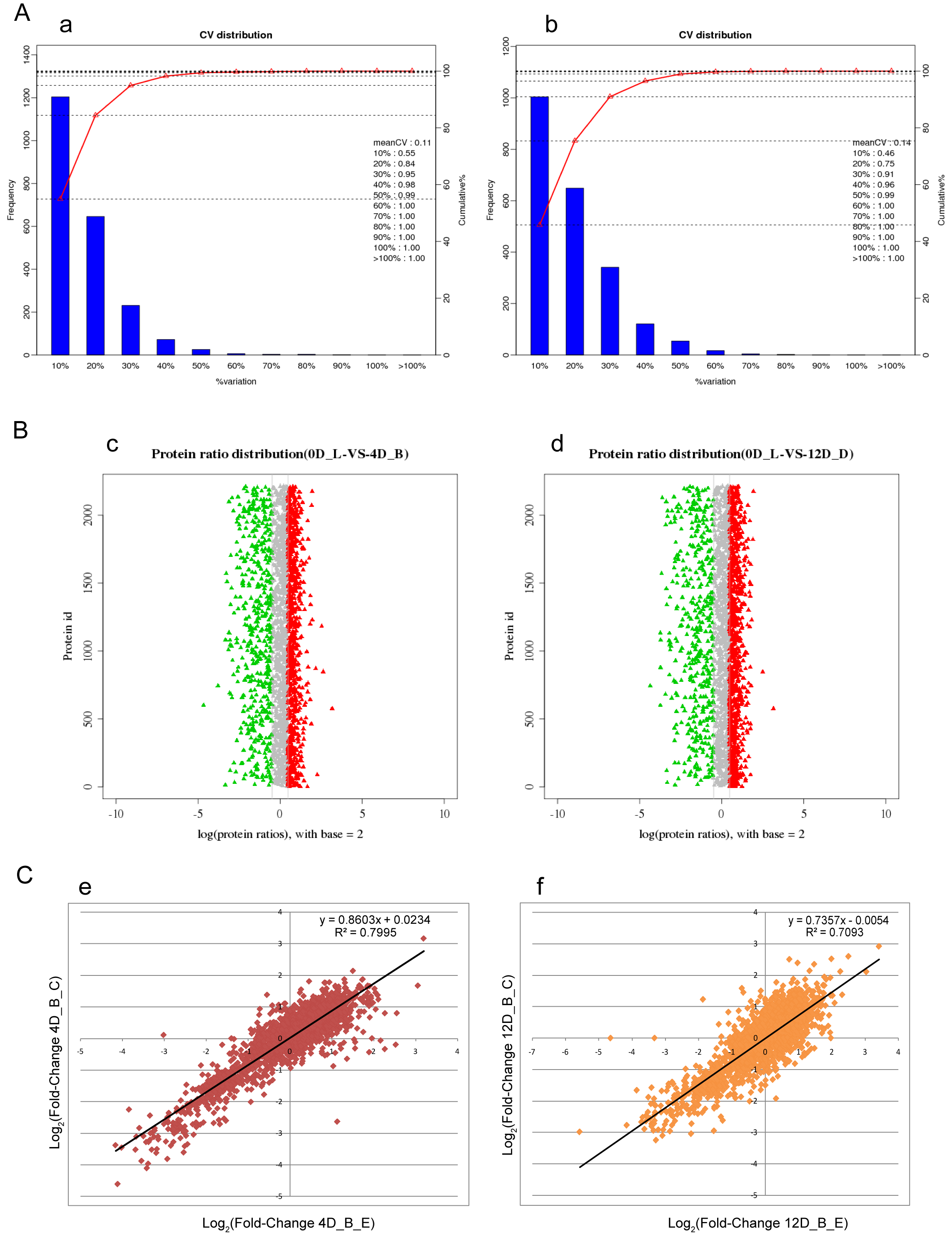

Supplement: Supplementary file 4 — Additional file 4. Congruence of evaluation among different data set. (A) CV distribution. a, 0D_L-VS-4D_B. b, 0D_L-VS-12D_D. (B) Protein ratio distribution. c, 0D_L-VS-4D_B. d, 0D_L-VS-12D_D. (C) Linear regression analysis based on the log2-transformed protein ratios. e, linear correlation between the two experiment replicates for 4D_B. f, linear correlation between the two experiment replicates for 12D_D. [file 12953_2019_153_MOESM4_ESM.tif]

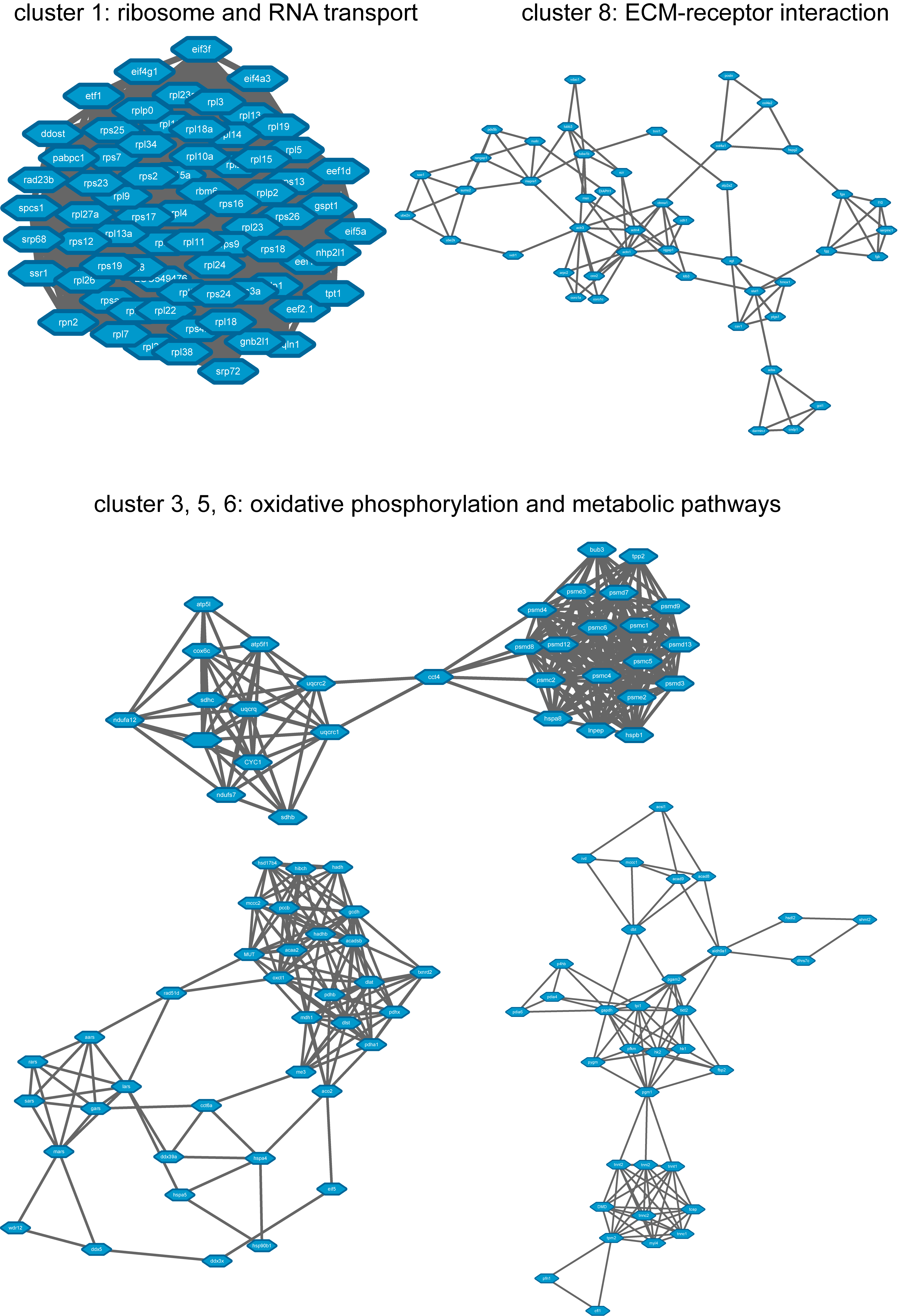

Supplement: Supplementary file 7 — Additional file 7. Some protein-protein interaction clusters that calculated by the MCODE software. [file 12953_2019_153_MOESM7_ESM.tif]
